# Hidden mysteries in ancient Egyptian paintings from the Theban Necropolis observed by in-situ XRF mapping

**DOI:** 10.1371/journal.pone.0287647

**Published:** 2023-07-12

**Authors:** Philippe Martinez, Matthias Alfeld, Catherine Defeyt, Hishaam Elleithy, Helen Glanville, Melinda Hartwig, François-Philippe Hocquet, Maguy Jaber, Pauline Martinetto, David Strivay, Philippe Walter

**Affiliations:** 1 Laboratoire d’archéologie moléculaire et structurale, Sorbonne Université, CNRS, Paris, France; 2 Mission Archéologique Française de Thèbes-Ouest (MAFTO), Centre d’Étude et de Documentation sur l’Ancienne Égypte, Ministère d’État des Antiquités, Cairo, Egypt; 3 Department of Materials Science and Engineering, Delft University of Technology, Delft, Netherlands; 4 Centre européen d’archéométrie, UR Art, Archéologie et Patrimoine, Université de Liège, Liège, Belgium; 5 Michael C. Carlos Museum of Art, Emory University, Atlanta, GA, United States; 6 Physique, Lumière Matière, CNRS/UGA UPR 2940, Institut Néel, Université Grenoble Alpes, Grenoble, France; Universita degli Studi di Milano, ITALY

## Abstract

The material study of ancient Egyptian paintings began with the advent of Egyptology during the 19th century. By the 1930s, a lot had already been sampled and described. The limited palette for example has been analysed from actual painted surfaces but also from pigments and painting tools retrieved on site. However, most of these studies took place in museums while the painted surfaces, preserved in funerary chapels and temples, remained somewhat estranged from this primary physical understanding. The artistic process has been also reconstructed, mainly from the information presented by unfinished monuments, showing surfaces at different stages of completion. A lot of this modern and theoretical reconstruction is, however, based on the usual archaeological guessing game that aims at filling the remaining blanks. Our interdisciplinary project has decided to experiment on-site with state-of-the-art portable analysis tools, avoiding any physical sampling, to see if our knowledge of the work of the ancient Egyptian painters and draughtsmen could be taken at a further stage, while based on physical quantification that could be seen as a stronger and more reliable foundation for a redefined scientific hypothesis. The use of XRF mapping has, for instance, been applied to a known case of correction by surface repaint, something that is supposedly rare in the ancient Egyptian formal artistic process, while another fully unexpected one was discovered during the analytic exploration of a royal representation. In both cases, the precise and readable imaging of the physical composition of the painted surface offers a renewed visual approach based of chemistry, that can be shared through a multi- and interdisciplinary approach. However, this also leads to a more complex description of pigment mixtures that could have multiple meanings, where the practical often leads towards the symbolic, and from there hopefully to a renewed definition of the use of colours in complex sets of ancient Egyptian representations. At this stage, though the progress in this on-site material assessment of ancient works of art definitely means astonishing progress, one humbly has to face the fact that these ancient treasures shall still retain part of their defining mysteries.

## Introduction

The Pharaonic Civilization offers the most extended cultural continuity of the ancient world. Its highly formalized painting style is easily recognized. This consistency mainly derives from aesthetic choices but also from an organized and regulated workflow that has been theoretically reconstructed by different authors [1–5], offering valuable insights into pigment use and painting techniques. It is assumed that the work started with a preliminary sketch drawn in red ochre on the plastered and smoothed wall, following general guidelines or even modular grids drawn on the wall surface. This was followed by the application of white or coloured backgrounds, at times leaving a reserve for pictorial details. Afterward, colour came, commonly with pigment mixtures and layered applications. As the last step, the final outline and details were drawn, mainly with red ochre. In a final and vital stage, paint spilling over the outline was overpainted with opaque white. One needs to be aware that this theoretical workflow is mainly based on the study of incomplete works in tomb chapels as well as artistic projects on ostraca, pottery sherds used for sketches dating to the 18th and 19th dynasties, a period perceived as the heyday of ancient Egyptian painting: these conclusions should then be generalized with care for all the other periods. It would be naïve, for instance, to assume that the techniques in use did not differ according to the paint substrate or even with each painter and “studio”.

Alterations to specific details of the paintings, during or after their execution, have also been reported but seem to remain rather seldom [6]. However, due to the rather opaque quality of the paint layers and the fact that most of the scientific inspections of these works of art have been carried out only through direct visual observation, this may very well be but the tip of the iceberg. Here we will study two cases of such alterations, trying to get an understanding of their raison-d’être, as well as of their modus operandi, through the use of chemical imaging techniques that finally give us access to the physical reality of what lies under the mesmerizing surface of these ancient Egyptian works of art. The question is thus to know whether there is more to see than what meets the eye.

This work is a part of a large project aimed, at first, at investigating tomb chapels from the Ramesside period (c. 1330—c. 1069 BC, 19th and 20th dynasties) in the Theban Necropolis. However, for the sake of understanding a technical approach that evolved over more than 3000 years, we expanded our research to nearby chapels from different periods and, initially, to those of the 18th Dynasty (around 1550–1350 BC). Located near the ancient city of Thebes, on the western bank of the River Nile, the necropolis reached its cultural heights during the New Kingdom (1549–1069 BC), setting the tombs of pharaohs and their families in the Valleys of the Kings and Queens. However, high-ranking officials (so-called nobles) were buried, for the most part, in a separate area of the necropolis, closer to the limit between the cultivated grounds and the desert foot-hills. Apart from the sealed subterranean vault containing the mummified dead, tombs featured chapels for the funerary offering cult, remaining open to the priests in charge and the family of the deceased, or even to more leisurely visitors in research of past beauty. The walls of these shrines were often decorated, representing the dead in his dutiful life on earth as well as his expectations for a bright future in the netherworld.

## Materials and methods

As of today, only a small number of tombs of the Theban Necropolis have been investigated in detail through the use of scientific techniques: the best-known examples are the tomb of Tutankhamun (KV 62) [7, 8], the tomb of Nefertari (wife of Ramses II, QV 66) [9] and the tomb of Menna (period of Thutmose IV, TT 69) [6, 10]. Samples taken from several tombs and a temple from the Theban Necropolis were investigated at Cairo University by various techniques to identify the pigments used [11–14]. During the 1980s, the Max Plank Institute of Heidelberg gathered more than 1000 samples emanating from diverse monuments [13, 15]. These and similar investigations [16, 17] provided broad insight into the pigments used in ancient Egypt [15]. These scientific results are generally in good agreement with the archaeological observations.

Recent years have nevertheless seen the development of chemical imaging techniques for the investigation of art objects, most prominently historical paintings [18–21]. Macro X-ray fluorescence imaging (MA-XRF) especially allowed new insights, as it provided elemental distribution images that are visually easier to grasp than the results of focused spot analysis. They also allow the demonstrative visualization of overpainted and altered pictorial details [22, 23]. For example, MA-XRF has also been used in combination with hyperspectral imaging for the investigation of a funerary Greco-Roman painted portrait dating from the 2nd century AD Egypt at the National Gallery in Washington, D.C. [24] or for the identification of the remnants of colours present on marble in Delphi (Greece) [25, 26]. The possibility of combining both methods with unsupervised machine learning (t-Stochastic Neighbour Embedding) has been demonstrated on Egyptian paintings [27].

XRF-imaging is well suited for the investigation of Egyptian paintings. As shown in Table 1, many pigments available to ancient Egyptian artists have elemental markers (in bold) detectable by XRF. Though limited when compared to later epochs, the palette gave the artist enough room for decision-making to achieve a specific colour effect by nuanced application of the desired shade of colour obtained through complex material mixing or layering.

**Table 1:** Common pigments of the 18th-20th dynasties of Egypt, based on [28].

Pigment	Colour	Composition
Hematite	Red	**Fe**_2_O_3_
Realgar	Red	**As**_4_ **S**_4_
Goethite	Yellow	*α*-**Fe**O(OH)
Orpiment	Yellow	**As**_2_ **S**_3_
Egyptian Blue (EB)	Blue	**CaCuSi**_4_O_10_
Egyptian Green	Green	EB+**Si**O_2_
Calcite	White	**Ca**CO_3_
Gypsum	White	**CaS**O_4.2_H_2_O
Anhydrite	White	**CaS**O_4_
Huntite	White	Mg_3_**Ca**(CO_3_)_4_
Carbon-based Black	Black	C

Elements detectable by XRF are highlighted in bold.

For our photogrammetric survey, images were taken with a 24 MPixel Sony NEX-7 camera. For the calculation of volumetric and spatial information Plexus by insightdigital.org (Institute for Study and Integration of Heritage Techniques, Berkeley, California) was used. Macrophotos were acquired using an OLYMPUS E-M5 Mark II, featuring 16.1 Mpixels, using a 60 mm macro-objective. Additional photos for documentation were taken with 5th generation iPhones.

For these studies, we used two portable XRF mapping instruments built by the LAMS (Sorbonne University-CNRS, France) and by the European Center for Archaeometry (Liège University, Belgium). These instruments described respectively in [26] and in [29, 30] are dedicated specifically for the on-site non-invasive investigation of heritage artifacts: this specification led to the production of lightweight devices, transportable in normal check-in luggage, that can be installed in narrow spaces and on uneven ground, while being battery operated if needed.

The two instruments consist of a measurement head with a X-ray tube with an Rh (3W) or Pd(5W) transmission anode (Moxtek, Orem, UT) and a SD-detector (25 mm^2^ or 70 mm^2^ active area, X-123FAST SDD, Amptek, Bedford, MA). The beam of the X-ray tube is collimated using a 0.8 mm collimator and impinged on the surface at normal angle. The fluorescence radiation is recorded under an angle of 58°. The distance between measurement head and wall is adjusted by a manual translation stage and during a scan the measurement head is moved over the surface of the wall by two motorized stages offering 30 cm travel range (Thorlabs, Newton, New Jersey, USA). Typical dwell times are between 0.2 and 1.0 seconds per pixel. Step sizes are between 0.5 and 1.2 millimetres. Elemental distribution images were calculated from the raw spectral XRF data by means of the Datamuncher [31] and PyMCA [32] software packages.

The two examples discussed in this publication are to be found respectively in the tomb chapels of Menna (an overseer of cadastral surveys under Amenhotep III, 1391–1353 B.C., TT 69) and Nakhtamun (Chief of the Altar in the Ramesseum, TT 341, possibly 20th Dynasty, circa 1100 B.C.). These tombs were selected for their excellent state of conservation and their accessibility. Though both monuments result from the elite culture of their time, they are quite unequal when confronted by modern science. While the paintings of the tomb of Menna are universally known as the apogee of ancient Egyptian painting, those of Nakhtamun remain underrated and simply inaccessible. Furthermore, the tomb of Menna was already studied by a part of our team more than ten years ago, through the use of X-ray fluorescence (XRF), diffuse reflectance UV-spectrometry, near-infrared (NIR) diffuse reflectance spectroscopy, and Raman spectroscopy [10]. A renewed study of these monuments could provide new insights into the more basic levels of craftsmanship. Awaiting a more complete presentation of our results, we would like to initially focus on the study of two important iconographic alterations.

## Results and discussions

### A third arm for Menna

**Fig 1:**
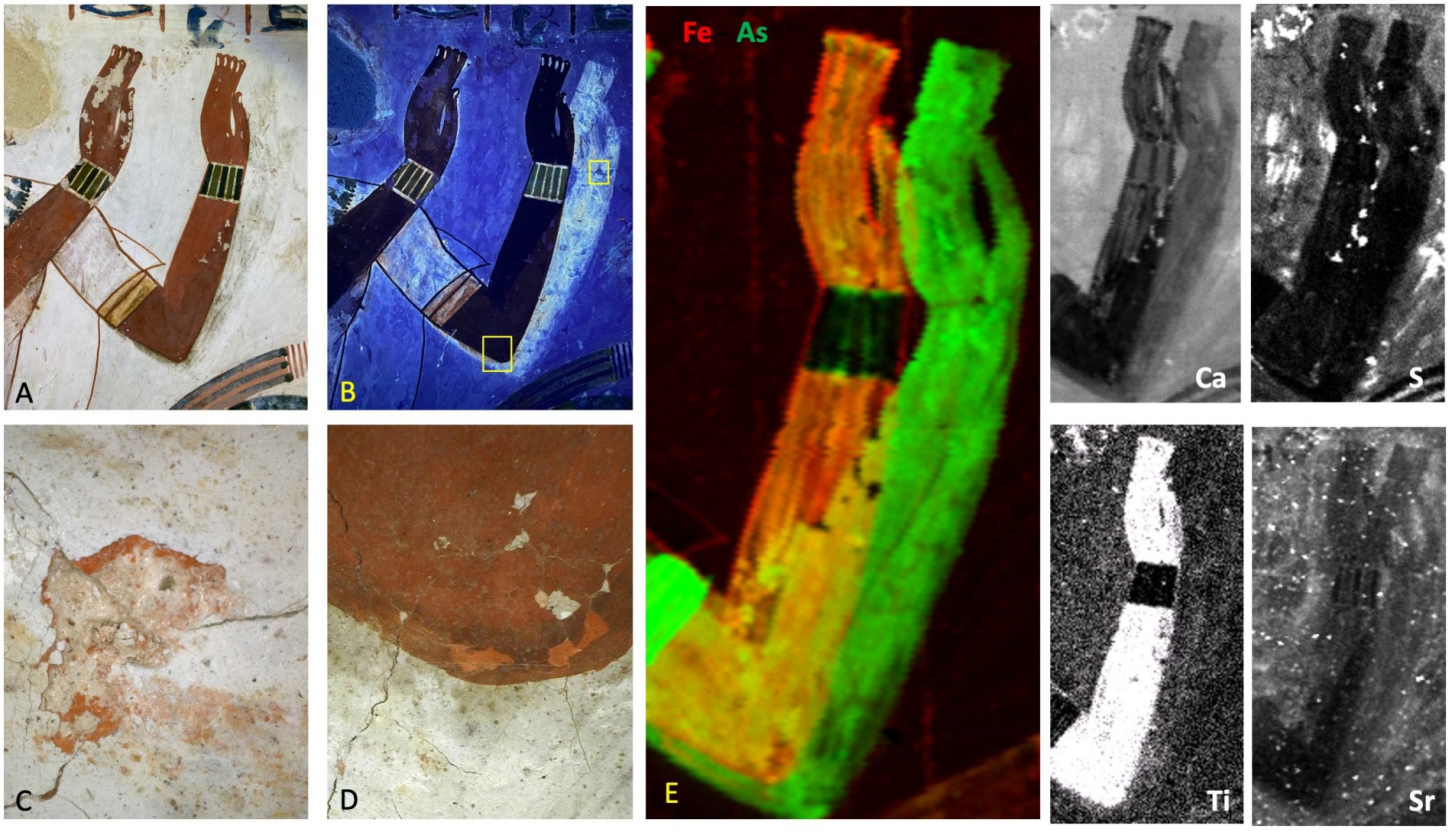
Observation of the painting of Menna. A: Visible picture. B: UV fluorescence picture. C and D: macro-photographs showing the colour of the first painting. E: MA-XRF study on an area of 12.8 x 22.8 cm2, scanned with a dwell time of 0.33 s/pixel and a step size of 1.0 mm. All distribution images are corresponding to the number of counts in the X-ray K-lines of the elements. All data are shown in S1 Fig).

The first alteration under discussion has been known since the discovery of the painted tomb chapel of Menna in 1888. It is indeed visible to the naked eye (Fig 1A). But that was surely not the case in ancient times. It has probably been made visible due to chemical interactions between the ancient materials and the migration of chemical compounds to the surface. The surface of the corrected area reacts with fluorescence under ultraviolet lighting (Fig 1B). This could indicate the presence of a specific organic binder or degradation product. This alteration is visible in a scene showing Menna and his wife adoring Osiris. This rite of adoration is visually made evident by the fact that Menna raises his two hands in front of his face. However, close observation of this area detects the presence of a third hand, somewhat hidden under the white surface layer that is used as a background. If this ghost hand and arm are thus easily detected, they remain frustratingly puzzling because the factual reasoning behind this alteration remains difficult to define precisely. From a strictly visual point of view, it has been described as resulting simply from an aesthetic issue. However, this is a modern judgment, and it remains rather difficult to justify what we could declare un-aesthetic would have appeared so to an ancient Egyptian. The change in the arm’s position is very slight, and it is difficult to say that it really changes anything to the stance of the worshipper.

The timing of the alteration remains in itself a problem. Was this correction applied just after the first composition was finished and soon seen as objectionable by either the painter himself or his patron? Or did some time elapse before this correction was deemed necessary and inescapable? The cohesion existing between the whitish overlay occluding the first arm and the general background would at least indicate that this correction was made during the initial stage of the tomb chapel decoration. Once this is stated, do we learn anything new from the two successive arms when studied through chemical mapping and the knowledge thus gained of the pigment composition of both successive images?

**Fig 2:**
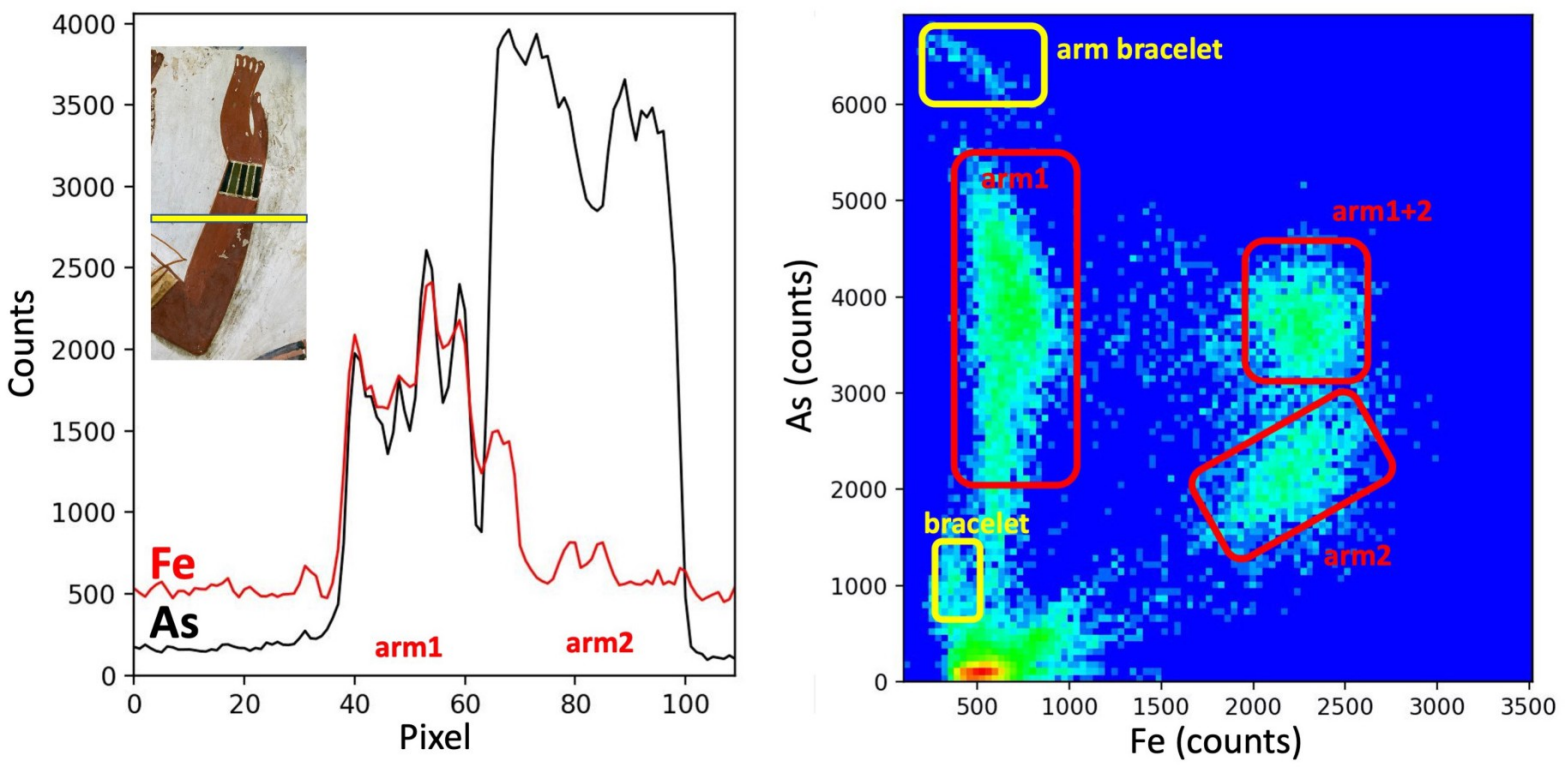
As and Fe distributions in Menna’s arm. A: XRF analysis along a line through the arm showing the evolution of FeK and AsK-lines (data from the MA-XRF in Fig 1). B: Scatterplot of Fe-K vs As-K lines showing their relative proportions in the different parts of the painting.

Considering that the correction should logically have been done at the same time with a similar technique, the XRF maps show surprisingly diverse pigment compositions (Fig 1E): the arsenic is more present in the area representing the first version of the arm than in the area showing the final arm. Fig 2 shows the scatter plot of Fe vs. As and the evolution of the signals of Fe and As along a horizontal line passing through the two versions. It is clearly visible that the first version was painted with an As-based pigment, resulting in a thick irregular layer, because the intensities of As-K lines are changing by more than a factor 2. The paint used for the second version is, on the contrary, a constant mixture of As and Fe-based pigments, as the intensities of As and Fe X-rays are clearly correlated. Obviously, when the second arm was painted over the first arm, the quantity of As is at its maximum.

This fact leads us to propose that the second version was simply laid upon the first one, remaining as an “undercoat.” This is surprising to our modern minds. If the painter needed to erase the original arm, it would have seemed wiser and more efficient to completely cover the deficient composition with an opaque white layer that would create a renewed background or underlayer. It is plausible that, over time, both pigments then reacted chemically [33] with each other or with an organic binder, leading to the formation of a flesh tone which is darker than that originally intended by the painter. That appears today somewhat unseemly. One could reason practically that the change should have been done on a later day, while the painter was simply and logically using a different mix of pigments. It is, however, interesting to note that, even taking into account the expedient aspect of the choice made by the painter, the physical presence of the As-based pigment would have been difficult to surmise visually, the iron-based ochre being rather opaque in its physical nature. One notices a definitely darker shading where the second arm overlays with the first, hidden, arm. The darker shade is thus surprising, as the ochre should be able to cover and hide the arsenic-based pigment present in the first arm.

Macrophotography of areas presenting defects in the white layer, revealing the first hidden version, and the red colour in the elbow representation (Fig 1C and 1D) make it possible to see the colour of the As-pigment used for the first arm. As it looks red-orange, it is probably made of realgar (As_4_S_4_). We can assume that it was also used for the second version but in this case mixed with a significant quantity of red ochre, rich in haematite (Fe_2_O_3_) possibly mixed with yellow ochre as described in [27]. The chemical maps show this ochre to be also rich in titanium (Ti) and silicon (Si) while containing a low concentration of potassium (K). Aluminium (Al) is not detected by our instrument. On the Fe map, the dashed lines visible behind the representation probably correspond to general guidelines drawn on the wall to set the columns for the hieroglyphic text.

Concerning the uses of pigments by the artist, it is also interesting to note that the gold bracelet on the arm was also painted with an As-based pigment of a yellow colour that was probably orpiment (As_2_S_3_). The distribution of Cu seems to confirm the usage of Egyptian Blue and Green and agrees well with the visual distribution of colours (S1 Fig)). The first arm was laid upon a limestone-based undercoat (calcium without sulfur in the XRF maps) that could be consistent with the use of a white colour probably made of calcium carbonate.

The fact that the two versions of the arm have different pigment compositions can give new clues as to the painting process. The paintings in the tomb of Menna were supposedly split between specialized draughtsmen and less skilled artisans: details in the brushwork in the tomb of Menna suggest that at least four different draughtsmen-scribes worked there [6, 34]. Betsy Bryan suggested that draughtsmen sketched and at times applied paint, but that those artisans of lower rank did not sketch [1]. The decoration of a tomb was, in general the work of several people working in parallel. But here we observe so many modifications that it is difficult to consider this segmented process was in use. This could suggest that the tomb was indeed constructed and decorated over a prolonged period of time with a significant number of people involved in the process of creating and modifying the wall paintings. Unfortunately, this remarkable encounter with the ghost of the painters at work does not teach us more about the reasoning at play in this formal, to us somewhat insignificant, visual change.

### A new necklace for Ramesses II

The tomb chapel of Nakhtamun dates to the Ramesside period. Because of a royal “portrait” specifically naming Ramesses II, it has usually been dated to his personal reign. This painting has also been discussed in detail because it shows a rare but precise representation of a pharaoh presenting a budding beard [35, 36]. Being seen as a visual symbol of grief, this portrait of Ramesses II was hypothetically linked to the death of his father and his rise to the throne. However, a closer examination of this image, in its own context, shows that Ramesses II then stands under a cult canopy, while the enthroned figure in front of him is clearly the god Ptah, not his deceased father Seti I. This being said, the artistic quality of the figure seemed interesting enough to have been painted by a skilled artist of the time. Upon closer inspection, it also presented an irregularity in terms of Egyptian iconography, Ramesses’s protrusive Adam’s apple, a detail that is interestingly never shown in ancient Egyptian art. We thus used MA-XRF in the hope of finding new insights on this painting.

**Fig 3:**
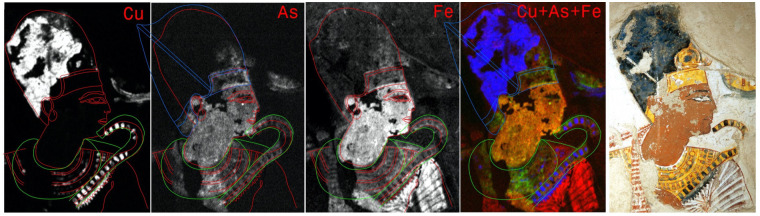
MA-XRF study of the painting of Ramesses II. MA-XRF study of the painting of Ramesses II on an area of 15.5 x 22.0 cm^2^, scanned with a dwell time of 0.23 s/pixel and a step size of 0.5 and 1.0 mm (horizontal and vertical, respectively). The area shown in detail was scanned with a step size of 0.5 and 0.5 mm and a dwell time between 0.5 and 0.7 s/pixel. All distribution images are shown in logarithmic scale of the number of counts in the X-ray K-lines of the elements. Sketch made with red line corresponds to the actual painting contour, green line corresponds to an earlier version and blue line corresponds to the khepresh original shape. All data are shown in S2 Fig).

Fig 3 shows the Cu, As and Fe chemical images of this portrait. Copper is characteristic of Egyptian Blue and Green. Some of the blue details of the collar that are barely visible today are clearly underlined by the Cu chemical image, because a few Egyptian Blue grains are still stuck on the plaster. The Cu also lies very well inside the red contours of the final painting, as it would be expected if the artists simply filled a sketched form.

Iron distribution agrees well with the different red and yellow tones. It is also present in the areas of the transparent clothing, where the chemical repartition map indicates that this effect was achieved by the application of a partly transparent white layer over a red one.

The Arsenic signal is mainly recorded in areas appearing as red and yellow on the painting, as if an As-based pigment was mixed with ochres, similarly to what has been described for the painting of Menna. However, they do not fit precisely with them, as seen for example in the area of the shoulder and for the sceptre. The sceptre seems indeed to have been painted in an initial stage considerably broader than in the final step. Unexpectedly, the first sceptre is also strangely not clearly separated from the face contour, as if entering in a collision with Ramesses’s chin. In this area, a close observation of the white surface reveals, in cracks, traces of a yellow layer, shining through. Here the pigment was likely to have been yellow orpiment (As_2_S_3_). Arsenic is also present in the outline of the golden forehead piece of the Egyptian crown. One must note that the yellow centre-piece of the same golden forehead piece is painted using solely a material deprived of any trace of As, a pigment that could very well be goethite. However, such an outline is not visible today and when comparing the As distribution with the registered image it can be seen that the bulk of the lower As line lies under the red skin flesh-tone of the face (not shown).

According to the distribution of Cu and Fe, we drew red lines on the chemical maps. They correspond to the painting as seen today. The outline of the As signals is, however, indicated by the green lines and seems to delineate an earlier version of the portrait.

As was the case in the representation of Menna’s arms, we observe here an interesting choice of pigments for the flesh tones. Arsenic is present, with Fe, only in the exposed skin of the pharaoh, but absent under the transparent clothes (S3 Fig). Macro-photographs of damaged areas in the face suggest the absence of a differently coloured underlayer in this region. However, the flesh tone of the royal face might result from the application of two different layers: the first one would reflect the use of red ochre, while the last layer shows an orange pigment mixture that could reflect the use of realgar. It could also be present in the reddish carnation of the lips.

Another eventuality could be that the skin tone was applied with a mixture of Fe and As containing paint. In this case, this technical approach could mean that the artist saw skin covered by cloth and exposed skin as two separate pictorial elements and thus painted them using different pigment mixtures. Whatever the plausible solution, this could also imply that the use of As containing pigment was not at all innocent.

We also observe the presence of As to the left of the head of Ramses behind the ear, but this cannot be associated meaningfully with a pictorial detail. However, it can be related to the fact that the khepresh-crown—as it is visible today—cannot be seen as exactly orthodox in its shape. It has been visibly altered and mostly in its rear part, giving it an elongated character that takes it out of the regular repertoire. The part that is behind the ear for example is very short. Thus, the As sensed behind the same ear could be the remnant of a former fuller representation of the golden band adorning the back of the khepresh. Blue lines which are traced on the figure probably correspond to its original shape.

At this stage, we attribute most of the distribution of As, which is not in agreement with the visible pictorial details, to an initial version of the royal figure executed partly with orpiment. It is interesting to note that all the insignia linked to Pharaonic kingship have thus been retouched: namely the khepresh-crown, the heka-sceptre and the wesekh-necklace that could have had initially a totally different shape. The sceptre seems to have been originally drawn as a straight line with a leftward curve at the end, unlike other Egyptian sceptres of this type, that usualy approach a circular shape towards the top. The same can be described for the golden forehead piece of the khepresh-crown. In this case also, the corners of the area were underlined by golden/yellow paint before partly overpainting it in the final execution. The degradation of orpiment into a more mobile chemical species has been observed in many artworks [37]. However, we do not believe this to be the cause for the spreading of As, as we did not observe a significant signal gradient towards the edges in the As distributions and would expect such a migration to be either an-isotropic or to follow gravity, and not upwards, as observed in the upper part of the sceptre.

In the current visible form, Ramses is depicted with a wesekh-necklace. A flat, circular piece of jewellery with an opening in the middle for the head, it is a common royal and divine adornment during the 19th dynasty, but also during most of the dynastic period. In the rounder shape visible in the As distribution, a shebyu-necklace could also be hypothetically recognized. These are formed by several voluminous chains of gold formed by large and heavy lenticular beads, that shape their maximum horizontal extension in the back, below the shoulder line. Shebyu collars, rather common during the reigns of Amenhotep III and Amenhotep IV-Akhenaten, are apparently unknown or not very common on the royal images of Ramesses II. They are, on the other hand, common in later royal portraits from the 20th dynasty. However, one must note that during the Ramesside period, this kind of necklace did not rise around the neck of the king as seems to be the case here. This may indicate that Nakhtamun did serve the dead and deified Ramses II rather than the living king. This could further mean that during the decoration of this chapel, the portrait of the pharaoh was first painted in the fashion of the 20th dynasty. Later, when the anachronistic or symbolically problematic nature of this piece of jewellery was recognized, the original composition was adjusted and simply repainted, in a fashion that made the former shebyu-necklace completely invisible to the naked eye. The hypothetical re-dating of the tomb to the 20th Dynasty, instead of the 19th Dynasty and the reign of Ramesses II, could be further strengthened by the rather elongated proportions of the figures in the chapel of Nakhtamun.

If this work hypothesis is interesting in itself, the significant retouching of the royal figure and its own dating remain very difficult to determine and to understand. We are in this case very far from the correction visible in the tomb of Menna, a correction that is also difficult to explain but that could result simply from a faulty preliminary composition. However, the complex changes appearing in what constitutes the truly royal iconography of the image of Ramesses II is surely connected to a change in symbolic meaning that unfortunately remains impossible to fathom. However, one can also note that seemingly meaningless corrections recently revealed on sculpted decoration at the Ramesseum, in places that are rather inaccessible even to the naked eye, show that we remain clearly ignorant of what was really important and significant for the ancient Egyptian eye and mind.

## Conclusion

Characterized by the Moderns as highly formalized, Egyptian art relied, on the one hand, on a structured workflow while on the other, it is also evident that workshop practices could vary significantly in the framework of this reconstructed and theoretical workflow. This inbred formalization should thus make corrections almost unheard of. However, two striking examples of this were studied in two selected tombs. If drawing general conclusions from two examples is in general ill-advised, these discoveries clearly call for a systematized and closer inspection of these painted surfaces using physicochemical characterization.

From a methodological perspective we have shown that the use of MA-XRF is not limited to experiments in laboratories and museums, but is also possible and highly rewarding in the field. To the authors’ best knowledge, no other mobile method is capable of detecting the complex superimposition of layers, while the sole use of XRF spot analysis might easily result in ill-advised and misguiding hypotheses, assuming the existence of pigment layers or mixtures instead of sketching. Consequently, we believe that XRF imaging may find a useful place in excavation campaigns in the same way it has now found its place in the investigation of historical paintings. This remains, however, still difficult due to the significant flow of complex data thus generated, a difficulty that could be dealt with by forthcoming technical progress.

## Supporting information

S1 Fig MA-XRF study of the painting of Menna’s arm. MA-XRF study on an area of 12.8 x 22.8 cm^2^, scanned with a dwell time of 0.33 s/pixel and a step size of 1.0 mm. All distribution images are corresponding to the number of counts in the X-ray K-lines of the elements. All data are shown in S1 Fig.
(TIF)

S2 Fig MA-XRF study of the painting of Ramesses II. MA-XRF study of the painting of Ramesses II on an area of 15.5 x 22.0 cm^2^, scanned with a dwell time of 0.23 s/pixel and a step size of 0.5 and 1.0 mm (horizontal and vertical, respectively). All distribution images are corresponding to the number of counts in the X-ray K-lines of the elements.
(TIF)

S3 Fig Fe and As correlation in Ramesses II. Correlations between Fe and As in two areas of the paintings of Ramesses II, displayed in red on the scatterplots restively to the areas displayed in purple on their respective chemical maps.
(TIF)

S1 File (ZIP)

S2 File (ZIP)

## References

[1] Bryan BM. Pharaonic painting through the New Kingdom. In: Lloyd AB, editor. A companion to Ancient Egypt—Vol. I. Oxford: Wiley-Blackwell; 2010. pp. 990–1007.

[2] Tiradritti F. Egyptian Wall Paintings. Paris: Citadelle-Mazenod; 2008.

[3] Hartwig M. Method in Ancient Egyptian painting. In: Angenot V and Tiradritti F, editors. Artists and colour in Ancient Egypt. Proceedings of the colloquium held in Montepulciano, August 22nd-24th, 2008. Montepulciano: Monografie Poliziane di Egittologia; 2016.

[4] Andreu-Lanoë G. L’art du contour: le dessin dans l’Égypte ancienne. Paris: Louvre éditions: Somogy; 2013.

[5] Iversen E, Shiabata Y. Canon and Proportions in Egyptian Art. Warminster: Aris and Phillips; 1975.

[6] Hartwig M. The tomb chapel of Menna (TT69). Cairo/New York: The American Research Center in Egypt; 2013.

[7] Wong L, Rickerby S, Phenix A, Rava A, Kamel R. Examination of the wall paintings in Tutankhamen’s Tomb: inconsistencies in original technology Stud Conserv 2012;57:322–330. doi: 10.1179/2047058412Y.0000000035

[8] Reeves NC. The Burial of Nefertiti? Amarna R Tombs Proj Occas Pap. 2015;1: 1–16.

[9] Corzo MA, Afshar M, editors. Art and eternity—The Nefertari wall painting conservation project 1986-1992. Los Angeles: The Getty Conservation Institute; 1993.

[10] Vandenabeele P, Garcia-Moreno R, Mathis F, Leterme K, Van Elslande E, Hocquet FP. Multi-disciplinary investigation of the tomb of Menna (TT69), Theban Necropolis, Egypt. Spectrochim Acta Part A 2009;73:546–552. doi: 10.1016/j.saa.2008.07.028 19010720

[11] Mahmoud HM, Papadopoulou L. Archaeometric analysis of pigments from the Tomb of Nakht-Djehuty (TT189), El-Qurna Necropolis, Upper Egypt. ArchéoSciences 2013; 37: 19–33. 10.4000/archeosciences.3967

[12] Mahmoud HM. Raman microscopic analysis of a multi-pigmented surface from the Theban Tomb (TT277), Luxor, Egypt. Acta Phys Pol A 2013; 123: 782–785. doi: 10.12693/APhysPolA.123.782

[13] Mahmoud HM. A multi-analytical approach for characterizing pigments from the tomb of Djhutyemhab (TT194), el-Qurna Necropolis, Upper Egypt. Archeometriai Muhely 2012; 9: 205–214.

[14] Mahmoud HM. A preliminary investigation of ancient pigments from the Mortuary Temple of Seti I, El-Qurna, Luxor, Egypt. Mediterr Archaeol Archaeom 2011; 11: 99–106.

[15] Blom-Boer I. The composition of the colour palette and the socio-economic role of pigments used in Egyptian painting. In: Thavapalan, S and Warburton, D, editors. The value of colour: material and economic aspects in the ancient world. Berlin: Topoi; 2019. pp. 231–255.

[16] Hussein MM, Kantiranis N, Ali M, Stratis J. Characterization of Ancient Egyptian wall paintings, the excavations of Cairo University at Saqqara. International Journal of Conservation Science 2011; 2 (3): 145–154.

[17] Augenti A, Greco C, Geismar H, Ovadia D, Ferraris M, Nicola M, et al. Archeologia invisibile. Torino: Museo Egizio; 2019.

[18] Alfeld M, de Viguerie L. Recent developments in spectroscopic imaging techniques for historical paintings—A review. Spectrochim Acta part B 2017;136:81–105. doi: 10.1016/j.sab.2017.08.003

[19] Alfeld M, Broekaert JAC. Mobile depth profiling and subsurface imaging techniques for historical paintings—A review. Spectrochim Acta part B 2013;88:211–230. doi: 10.1016/j.sab.2013.07.009

[20] Alfeld M, Vaz Pedroso J, van Eikema Hommes M, van der Snickt G, Tauber G, Blaas J. et al. A mobile instrument for in situ scanning macro-XRF investigation of historical paintings. J Anal At Spectrom 2013; 28: 760–767. doi: 10.1039/c3ja30341a

[21] Alberti R, Frizzi T, Bombelli L, Gironda M, Aresi N, Rosi F. et al. CRONO: a fast and reconfigurable macro X-ray fluorescence scanner for in-situ investigations of polychrome surfaces. X-Ray Spectrom 2017; 46: 297–302. doi: 10.1002/xrs.2741

[22] Galli A, Caccia M, Alberti R, Bonizzoni L, Aresi N, Frizzi T. et al., Discovering the material palette of the artist: a p-XRF stratigraphic study of the Giotto panel “God the Father with Angels”. X-Ray Spectrom 2017; 46: 435–441. doi: 10.1002/xrs.2751

[23] Alfeld M, Siddons DP, Janssens K, Dik J, Woll A, Kirkham R. et al. Visualizing the 17th century underpainting in Portrait of an Old Man by Rembrandt van Rijn using synchrotron-based scanning macro-XRF. Appl Phys A 2013; 111: 157–164. doi: 10.1007/s00339-012-7490-5

[24] Delaney JK, Dooley KA, Radpour R, Kakoulli I. Macroscale multimodal imaging reveals ancient painting production technology and the vogue in Greco-Roman Egypt. Scientific Report 2017; 7: 15509. doi: 10.1038/s41598-017-15743-5 29138483 PMC5686187

[25] Alfeld M, de Viguerie L, Devogelaere J, Muliez M. MA-XRF and hyperspectral reflectance imaging for visualizing traces of antique polychromy on the Frieze of the Siphnian Treasury. Microchem J 2018;141:395–403. doi: 10.1016/j.microc.2018.05.050

[26] Alfeld M, Cain K, Martinez P, Mulliez M, Jockey P, Walter P. The Eye of the Medusa—XRF imaging reveals unknown traces of antique polychromy. Anal Chem 2016; 89: 1493–1500. doi: 10.1021/acs.analchem.6b03179 27992167

[27] Alfeld M, Pedetti S, Martinez P, Walter P. Joint data treatment for Vis–NIR reflectance imaging spectroscopy and XRF imaging acquired in the Theban Necropolis in Egypt by data fusion and t-SNE. Comptes Rendus Phys 2018; 19: 625–635. doi: 10.1016/j.crhy.2018.08.004

[28] Scott DA. A review of ancient Egyptian pigments and cosmetics. Stud Conserv 2016;61:185–202. doi: 10.1179/2047058414Y.0000000162

[29] Hocquet PH, Calvo del Castillo H, Cervera Xicotencatl A, Bourgeois C, Oger O, Marchal A. et al. Elemental 2D imaging of paintings with a mobile EDXRF system. Anal Bioanal Chem 2011; 399(9): 3109–3116. doi: 10.1007/s00216-010-4281-8 20953768

[30] Strivay D, Clar M, Rakkaa S, Hocquet FP, Defeyt C. Development of a translation stage for in situ noninvasive analysis and high-resolution imaging. Appl Phys A 2016; 122(11): 950. doi: 10.1007/s00339-016-0476-y

[31] Alfeld M, Janssens K. Strategies for processing megapixel X-ray fluorescence hyperspectral data: a case study on a version of Caravaggio’s painting Supper at Emmaus. J Anal At Spectrom 2015; 30: 777–789. doi: 10.1039/C4JA00387J

[32] Solé VA, Papillon E, Cotte M, Walter P, Susini J. A multiplatform code for the analysis of energy-dispersive X-ray fluorescence spectra. Spectrochim Acta Part B 2007; 62: 63–68. doi: 10.1016/j.sab.2006.12.002

[33] Keune K, Mass J, Mehta A, Church J, Meirer F. Analytical imaging studies of the migration of degraded orpiment, realgar, and emerald green pigments in historic paintings and related conservation issues. Heritage Science 2016; 4/10. 10.1186/s40494-016-0078-1

[34] Kozloff AP. Theban tomb paintings from the reign of Amenhotep III: Problems in iconography and chronology. In: Berman L, editor. The Art of Amenhotep III: Art Historical Analysis. Cleveland: The Cleveland Museum of Art; 1990. pp.55–64.

[35] Desroches-Noblecourt C. Une coutume égyptienne méconnue. BIFAO 1947; 45: 185–232.

[36] Minault-Gout A. Ostracon figure: profil de pharaon. In: Andreu G, editor. Les artistes de Pharaon. Paris: RMN; 2002. p172.

[37] Zidan EH, Mosca S, Bellei S, Frizzi T, Gironda M, El-Rifai I. et al. In situ imaging, elemental and molecular spectroscopy for the analysis of the construction and painting of a Late Period coffin at the Egyptian Museum of Cairo. Measurement 2018; 118: 379–386. doi: 10.1016/j.measurement.2017.11.055

