# Supplementary figures and images for: Hidden mysteries in ancient Egyptian paintings from the Theban Necropolis observed by in-situ XRF mapping
Source: PLoS One. 2023 Jul 12;18(7):e0287647. doi: 10.1371/journal.pone.0287647 (PMC10337869; doi:10.1371/journal.pone.0287647)

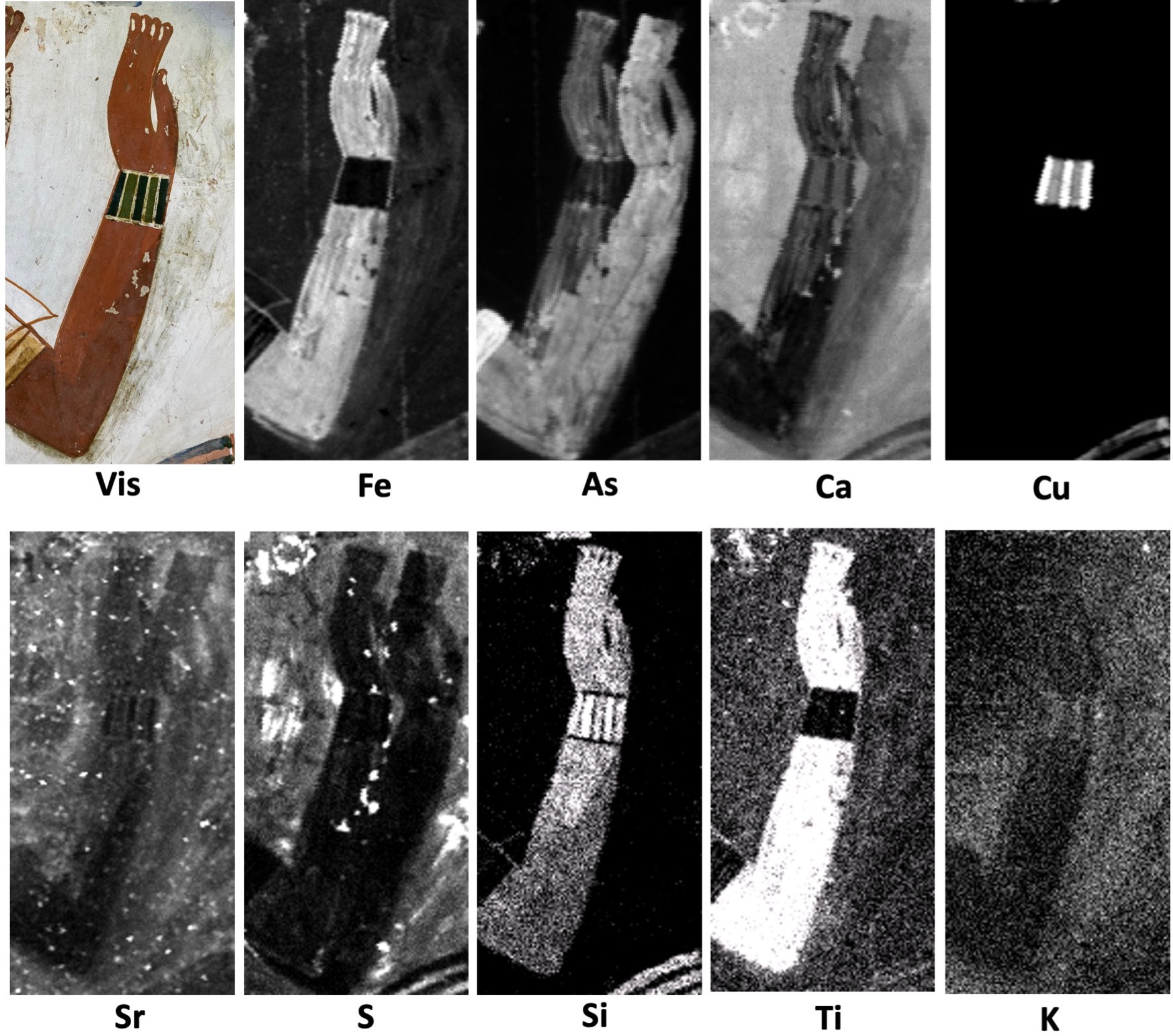

Supplement: S1 Fig — MA-XRF study on an area of 12.8 x 22.8 cm2, scanned with a dwell time of 0.33 s/pixel and a step size of 1.0 mm. All distribution images are corresponding to the number of counts in the X-ray K-lines of the elements. All data are shown in S1 Fig. (TIF) [file pone.0287647.s001.tif]

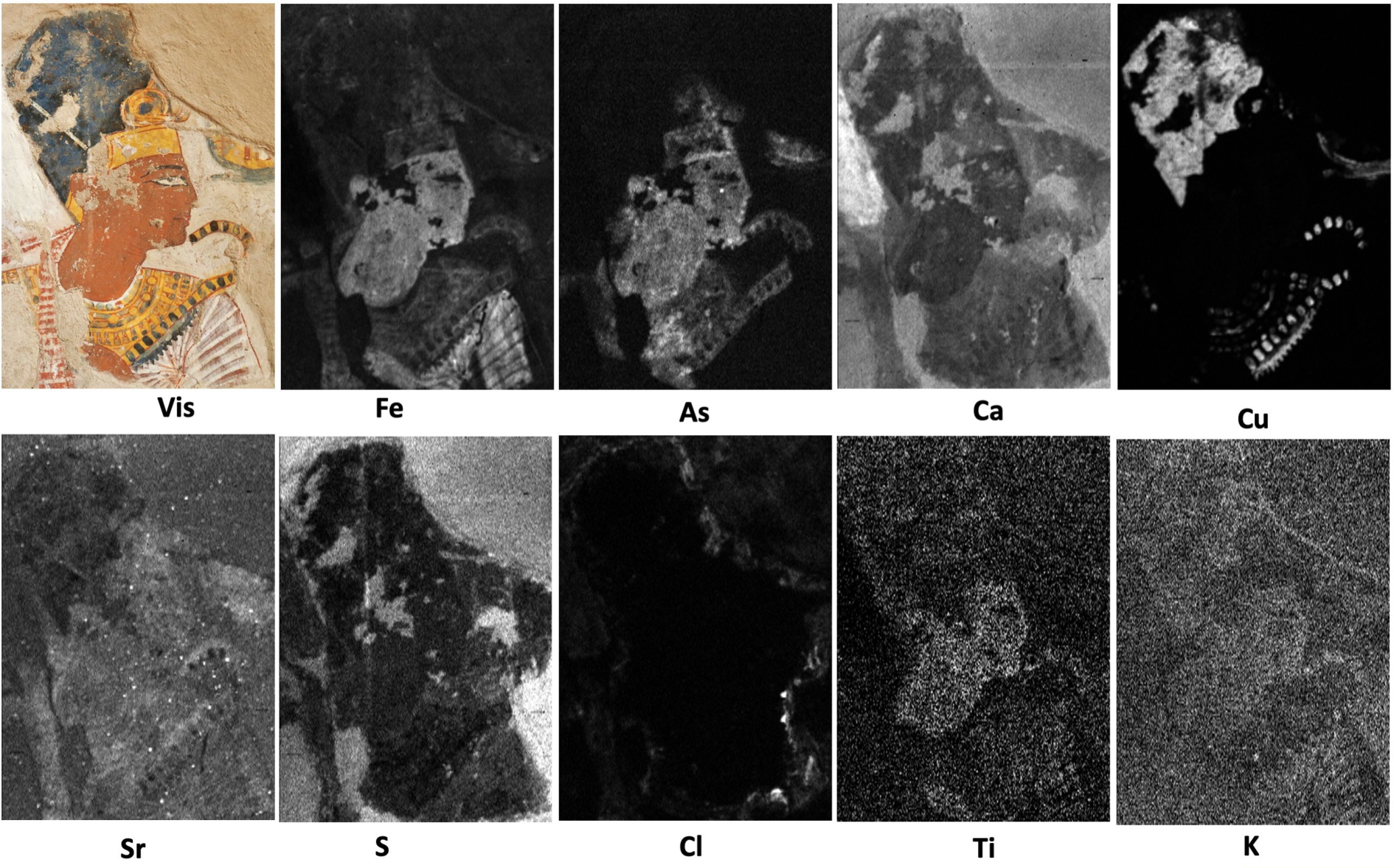

Supplement: S2 Fig — MA-XRF study of the painting of Ramesses II on an area of 15.5 x 22.0 cm2, scanned with a dwell time of 0.23 s/pixel and a step size of 0.5 and 1.0 mm (horizontal and vertical, respectively). All distribution images are corresponding to the number of counts in the X-ray K-lines of the elements. (TIF) [file pone.0287647.s002.tif]

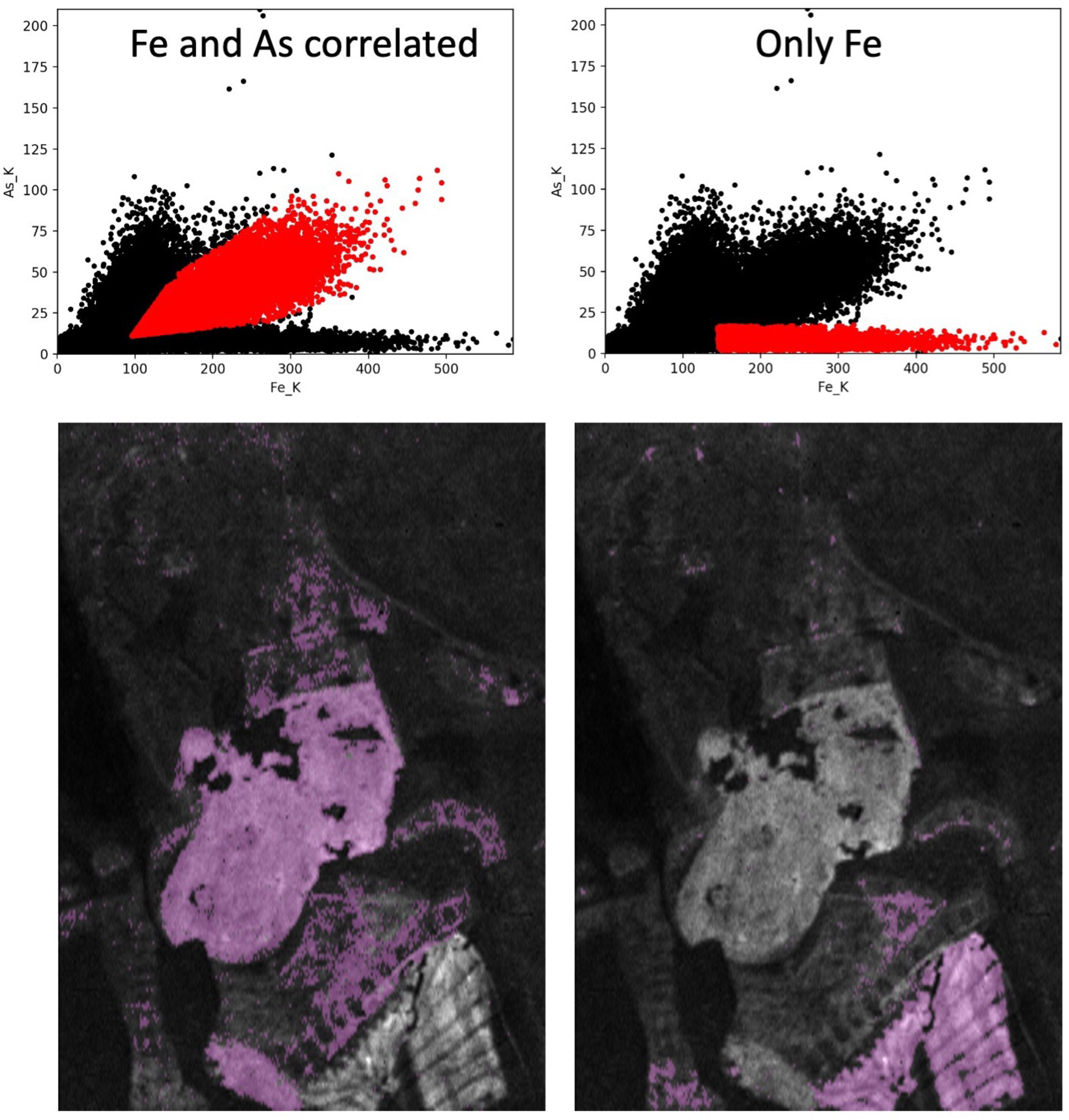

Supplement: S3 Fig — Correlations between Fe and As in two areas of the paintings of Ramesses II, displayed in red on the scatterplots restively to the areas displayed in purple on their respective chemical maps. (TIF) [file pone.0287647.s003.tif]
